# Synergizing reproductive efficiency and growth performance: A large-scale evaluation of Dorper × Garut crossbreeding in Indonesian sheep

**DOI:** 10.14202/vetworld.2025.2934-2944

**Published:** 2025-10-08

**Authors:** Zaenab Nurul Jannah, Panjono Panjono, Amir Husaini Karim Amrullah, Bayu Andri Atmoko, Siti Aslimah, Adi Tiya Warman, Mohammad Firdaus Hudaya, Besse Tenri Nurul Hikmah, Asep Sudarman, Alek Ibrahim

**Affiliations:** 1 Department of Animal Production, Faculty of Animal Science, Universitas Gadjah Mada, Jl. Fauna No. 3, Bulaksumur, Yogyakarta, 55281, Indonesia; 2 Department of Animal Science, Faculty of Agriculture, University of Bengkulu, Jl. WR Supratman Kandang Limun, Bengkulu, 38122, Indonesia; 3 Research Center for Animal Husbandry, Research Organization of Agriculture and Food, National Research and Innovation Agency (BRIN), Jalan Raya Jakarta-Bogor KM 46, Cibinong Science Center, Cibinong, Bogor, 16911, Indonesia; 4 Animal Science Study Program, Faculty of Pharmacy, Health, and Science, Universitas Muhammadiyah Kuningan, Jl. Raya Pangeran Adipati No. D4, Kuningan, 45552, Indonesia; 5 Department of Animal Bioscience, Faculty of Food Security, Universitas Negeri Surabaya, Kampus Unesa 3, Jl. Prof. Dr. Moestopo No.4, Surabaya, 60131, Indonesia; 6 Department of Animal Nutrition and Feed Technology, Faculty of Animal Science, IPB University, Jl. Raya Dramaga Kampus IPB Dramaga Bogor, Bogor, 16680, Indonesia

**Keywords:** Dorper sheep, Garut sheep, Crossbreeding, Lambing interval, Productivity index, Indonesia

## Abstract

**Background and Aim:**

Sheep farming is a vital component of Indonesia’s agricultural economy, where the demand for meat continues to rise. Indigenous Garut sheep are well-adapted to local environments and are known for their high reproductive efficiency, but they have low growth rates. Conversely, Dorper sheep are renowned for their rapid growth and carcass quality, but they exhibit longer lambing intervals under tropical conditions. Crossbreeding offers a strategy to combine the strengths of both breeds. This study aimed to evaluate maternal reproductive performance and pre-weaning growth traits in purebred Dorper, Garut, and Dorper × Garut crossbred sheep under a commercial breeding system in Indonesia.

**Materials and Methods:**

A retrospective observational study was conducted on 1,744 ewes (1,498 Garut, 209 F1 Dorper × Garut, and 93 Dorper) and 3,248 lambs (2,846 F1 Dorper × Garut, 253 B1 backcrosses, and 149 Dorper) from a commercial enterprise in West Java. Data included lambing interval, litter size, birth weight, weaning weight, pre-weaning mortality, average daily gain (ADG), reproductive index, and productivity index. Statistical analyses employed one-way analysis of variance with Duncan’s multiple range test for *post hoc* comparisons.

**Results:**

F1 Dorper × Garut crossbred ewes demonstrated significantly shorter lambing intervals (206.65 ± 2.75 days) than pure Dorper (265.66 ± 1.14 days), comparable to Garut ewes (209.10 ± 1.08 days). However, Garut ewes had superior litter size (1.77 ± 0.18) relative to both crossbred (1.33 ± 0.04) and Dorper ewes (1.30 ± 0.42). In growth performance, Dorper lambs excelled in birth weight (3.35 ± 0.07 kg), weaning weight (23.93 ± 0.57 kg), and ADG (203.88 ± 4.65 g/day). F1 Dorper × Garut lambs showed significantly higher weaning weight (19.48 ± 0.35 kg) and ADG (165.34 ± 2.95 g/day) compared with Garut lambs (15.36 ± 0.10 kg; 130.47 ± 0.83 g/day).

**Conclusion:**

F1 Dorper × Garut crossbreeding synergizes Garut’s reproductive efficiency with Dorper’s growth performance, yielding crossbreds well-suited for tropical meat production. While Garut maintains a prolificacy advantage, F1 crossbreds deliver improved pre-weaning growth, supporting their use in commercial fattening programs. Maintaining pure Garut flocks for breeding and employing F1 crossbreds for production may enhance productivity and sustainability in Indonesia. Future research should assess carcass traits, multigenerational crossbreeding, and economic feasibility.

## INTRODUCTION

The livestock sector is a cornerstone of Indonesia’s agricultural economy, with sheep farming playing an essential role in sustaining rural livelihoods and meeting the nation’s growing demand for meat. According to Badan Pusat Statistik-Statistics Indonesia (2023), the national sheep population rose from 16.8 million in 2022 to 17.5 million in 2023, reflecting notable sectoral growth [1]. Beyond its economic contribution, sheep farming also carries deep cultural and religious significance [2]. With a Muslim population of approximately 236 million, Indonesia generates substantial demand for sheep. For example, if half of the 57 million Muslim households were to sacrifice a sheep or goat during Eid al-Adha, an estimated 14.25 million animals would be required. Similarly, the annual demand for sheep and goats for *aqiqah*, a religious celebration marking the birth of a child, amounts to roughly 5.85 million animals [3]. These conditions underscore the urgency of strategies aimed at enhancing sheep and lamb production to meet both cultural and commercial needs.

Garut sheep, a native Indonesian breed, are well adapted to local environments and valued for their early sexual maturity and high reproductive efficiency. They can thrive under suboptimal conditions, efficiently utilizing low-quality forage while maintaining strong fertility and prolificacy [4, 5]. Nevertheless, the breed faces limitations such as slow growth, low slaughter weight, and modest carcass yield, which reduce its commercial competitiveness in large-scale production systems [6]. In contrast, Dorper sheep, developed in South Africa, are renowned for rapid growth, high carcass quality, and efficient feed utilization [7, 8]. Their superior meat production traits make them attractive for commercial farming [9, 10], though their imported status in Indonesia poses challenges, including reduced adaptability to the tropical climate and potential constraints on reproductive performance [6].

Crossbreeding Garut with Dorper sheep offers a promising solution, combining the environmental resilience and reproductive strengths of Garut with the growth and carcass advantages of Dorper. The resulting heterosis, or hybrid vigor, typically improves growth rate, weaning weight, and overall productivity in F1 crossbreds [6, 11]. Indeed, previous studies by Athifa *et al*. [6], Mohammed *et al*. [12], and Găvojdian *et al*. [13] have demonstrated that Dorper × Garut F1 lambs exhibit faster growth and higher viability compared to purebreds, highlighting a a strong potential for genetic improvement within Indonesia’s sheep industry. Moreover, hybrid vigor has been linked to improved reproductive traits, including greater fertility, larger litter sizes, and enhanced survivability, key factors in ensuring the economic sustainability of sheep farming [14, 15].

Despite the recognized advantages of crossbreeding programs in sheep, there remains limited large-scale, empirical evidence regarding the reproductive and growth performance of Dorper × Garut crossbreds under Indonesian commercial production systems. Most existing studies on this cross have been conducted at smallholder or research-station levels, often with relatively small sample sizes and shorter observation periods [6, 12, 13]. These studies provide valuable insights but may not fully capture the variability and challenges present in large, commercial flocks operating under diverse environmental conditions. Furthermore, while Garut sheep are widely acknowledged for their prolificacy and adaptability, and Dorper sheep for their superior growth traits, there has been insufficient direct comparison of reproductive traits (such as lambing interval, litter size, and reproductive index) and pre-weaning growth performance (including birth weight, weaning weight, and average daily gain [ADG]) across purebred Garut, purebred Dorper, and their crossbreds. The lack of comprehensive, field-based data limits the ability of breeders and policymakers to design evidence-based crossbreeding strategies tailored for Indonesian environments.

This study was designed to address these gaps by conducting the first large-scale comparative evaluation of reproductive and pre-weaning growth traits in purebred Garut, purebred Dorper, and F1 Dorper × Garut crossbred sheep within a commercial production setting in West Java, Indonesia. Specifically, the study aimed to (1) assess maternal reproductive performance through parameters such as lambing interval, litter size, reproductive index, and productivity index and (2) evaluate pre-weaning growth traits, including birth weight, weaning weight, and ADG. By integrating reproductive efficiency with growth performance, this research provides a comprehensive assessment of the potential advantages and trade-offs of Dorper × Garut crossbreeding. Ultimately, the findings are expected to inform sustainable breeding policies, optimize production strategies, and support the development of a more competitive sheep industry in Indonesia.

## MATERIALS AND METHODS

### Ethical approval

All procedures were approved by the Ethics Committee of the Faculty of Veterinary Medicine, Universitas Gadjah Mada (Protocol No. 0037/EC-FKH/Eks./2020).

### Study period and location

This study was conducted from May to November 2024. During this period, the research activities involved the direct observation and retrospective extraction of performance data from the farm’s database and a concurrent on-site evaluation. This evaluation was performed to systematically document current farm management practices, nutritional programs, and environmental conditions. As part of the on-site assessment, microclimatic data were monitored daily using Elitech thermohygrometers (Elitech Technology Inc., USA), recording an average ambient temperature of 25.62°C and a relative humidity of 79%. These environmental conditions support the operation of sheep breeding.

The study was centered on a commercial sheep breeding enterprise in the highlands of Malangbong, Garut Regency, West Java, Indonesia (Latitude: 7.024036° S, Longitude: 108.083404° E). Established in 2002, commercial sheep breeding manages a flock of over 4,000 sheep and focuses on the development of two primary breeds: indigenous Garut sheep, officially recognized by the Minister of Agriculture Decree as local sheep genetic resources [16], and Dorper sheep, imported from Australia in 2017. The Dorper sheep have also been approved for breeding in Indonesia as an introduced (exotic) sheep genetic resource as per the Decree of the Minister of Agriculture [17]. The farm operates at an altitude ranging from 675 to 720 m above sea level.

### Study design

This retrospective observational study was designed to evaluate the performance of different sheep breeds under a commercial production system. The core of the study involved analyzing a historical dataset of production records, which were anonymized and extracted from the farm’s database. No alterations to animal management or invasive procedures were performed by the researchers. A subsequent on-site observational period was conducted solely to document the operational protocols.

### Breeding strategy and animal groups

The study used sheep bred and raised at a commercial sheep breeding enterprise under standardized company breeding protocols. The farm has established a structured crossbreeding program involving both imported and in-house-bred purebred Dorper sheep and indigenous Indonesian Garut sheep. Over time, this program has produced F1 Dorper × Garut crossbreds (first-generation hybrids). While the crossbreeding program remains ongoing and continues to be refined for long-term genetic improvement, this study focuses on three defined mating groups to evaluate their reproductive and pre-weaning growth performance.

**Figure 1:**
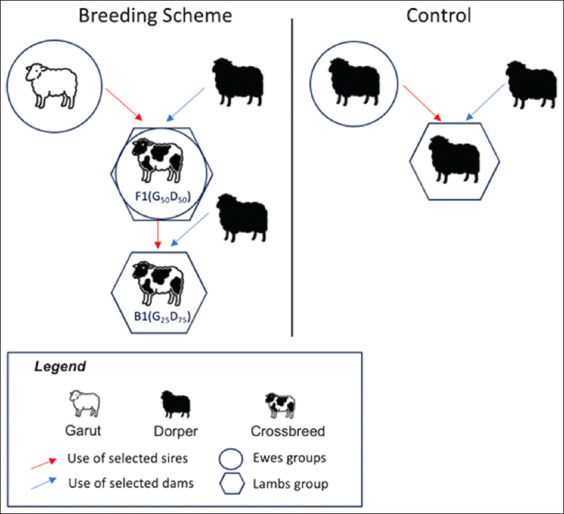
Illustration of the crossbreeding scheme between Garut and Dorper sheep in this study.

The breeding schemes used in this study are illustrated in Figure 1. The three distinct mating groups were as follows:

1. Purebred Garut ewes (n = 1,498) were mated with purebred Dorper rams to produce F1 Dorper × Garut crossbreds (n = 2,846), which comprised 50% Garut and 50% Dorper genetics.
2. F1 Dorper × Garut females were retained as breeding ewes (n = 209) and backcrossed with purebred Dorper rams to produce B1 Dorper × Garut crossbreds (n = 253), which comprised 25% Garut and 75% Dorper genetics.
3. The control group consisted of purebred Dorper ewes (n = 93) mated with purebred Dorper rams to produce purebred Dorper lambs (n = 149) representing the imported breed and their purebred offspring.

### Animal management and breeding protocols

The commercial sheep breeding enterprise implements a structured breeding management system to enhance reproductive efficiency and growth performance. Ewes selected for breeding must be at least 8 months old, weigh a minimum of 30 kg, and be in good health. Dorper rams must be at least 1.5 years old, weigh over 60 kg, and pass evaluation for libido and semen quality.

Natural mating was conducted in elevated colony pens measuring 5 × 9 m², each accommodating one Dorper ram and 11–15 ewes and providing 3 m² of floor space per animal. The mating period lasted 60 days, allowing adequate time to accommodate natural variations in ewes’ estrous cycles and maximize conception rates without the use of hormonal synchronization. A total of 60 Dorper rams were used across the breeding groups. Pens are equipped with elevated (2 m) slatted floors (with a 2–3 cm gap) constructed from wooden materials, adequate lighting (the pens received ample daily sunlight from 08:00 am to 05:00 pm), natural ventilation (partially open walls with a height of 1 m), and feeding and drinking facilities.

To reduce the risk of false-positive results associated with early gestation, pregnancy diagnosis was performed using ultrasound scanning with a 5 MHz Draminski ultrasound scanner probe transducer (Draminski S.A., Poland) at the end of the mating period. Ewes confirmed to be pregnant were transferred to gestation pens for a period of 60–90 days, whereas non-pregnant ewes were returned to the next mating cycle. Post-lambing management included close monitoring of placental expulsion, maternal behavior, and lamb nursing to ensure neonatal viability. Ewes received anthelmintic treatment (albendazole) and grooming procedures, such as shearing, bathing, and hoof trimming, between 3–7 days postpartum. Dorper rams were rested for 30 days following each mating cycle to allow recovery and reconditioning. The ewes and their lambs were returned to colony pens 30 days after lambing, and the breeding cycle was repeated every 60 days postpartum.

Lambs were weaned at 90 days of age. During the pre-weaning period, creep feeders were provided to provide adequate nutritional intake. Twin and triplet lambs were observed to ensure equitable nursing and reduce early-life growth variation. Feed was provided twice daily, and clean drinking water was provided ad libitum through automatic watering systems. Nutritional management followed the internal standards of commercial sheep breeding enterprise in both quality and quantity, with diets tailored to the physiological needs of the animals. The feed amount was adjusted to the nutritional needs of the sheep following the National Research Council nutritional standards, namely 2.5%–3.5% dry matter based on body weight [18]. The diet consists of fresh forage, specifically Pakchong grass (*Pennisetum purpureum* cv. Pakchong), supplemented with a house-made commercial concentrate formulated for breeding purposes (Table 1).

**Table 1:** Nutritional composition of feed ingredients.

Feed ingredient	Nutritional composition (%)						

	DM	CP	EE	CF	Ash	NFE	TDN
*Pennisetum purpureum* cv. Pakchong (Pakchong grass)	10.99	6.21	1.97	30.84	11.65	43.07	60.37
Commercial concentrate	87.16	14.36	4.11	17.71	8.56	63.67	72.44

DM = Dry matter, CP = Crude protein, EE = Ether extract, CF = Crude fiber, NFE = Nitrogen-free extract, TDN = Total digestible nutrients

### Data extraction and performance indicators

This study integrated primary data from direct measurements and observations with secondary data from the production data record. This study used retrospective production data extracted from the digital records of a commercial sheep breeding enterprise from January 2019 to December 2023. All procedures related to animal management and data recording were conducted by trained farm staff during routine farm operations. Extracted parameters included lambing date, litter size, lamb sex, birth weight, weaning weight, and pre-weaning mortality.

Lamb birth weights were recorded within 24 h using a calibrated livestock scale (Hitachi, Japan, 300 kg capacity, 0.01 kg precision). Weaning weights were recorded at 90 days of age in accordance with the standard weaning protocol of the farm. The lambing interval was calculated as the duration between two successive parturitions from the same ewe. Pre-weaning mortality was determined by comparing the number of lambs born to those surviving until weaning.

Based on the extracted data, the following performance indicators were calculated:


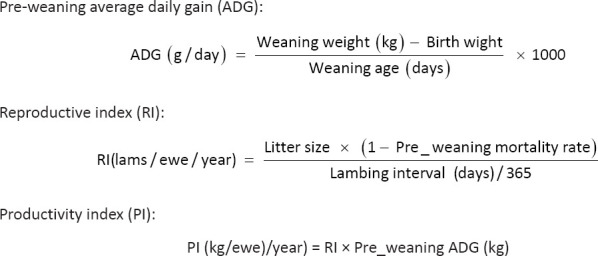


### Statistical analysis

All data were analyzed using the Statistical Package for the Social Sciences version 25.0 (IBM Corp., NY, USA). Before analysis, the Kolmogorov–Smirnov test was used to evaluate data distribution for normality, and Levene’s test was used to assess homogeneity of variances. Differences in reproductive and pre-weaning growth performance among the three genetic groups (F1, Garut, and Dorper) were assessed using one-way analysis of variance (ANOVA). If the ANOVA result was significant (p < 0.05), Duncan’s multiple range test was used to perform *post hoc* comparisons to identify specific differences between group means.

## RESULTS

### Reproductive performance

Significant differences in reproductive performance were observed among purebred Dorper, Garut, and their crossbreeds (Table 2). The lambing interval was significantly shorter (p < 0.01) in F1 Dorper × Garut crossbred ewes (206.65 ± 2.75 days) than in Dorper ewes (265.66 ± 1.14 days), whereas no difference was detected between F1 Dorper × Garut crossbred and Garut ewes (209.10 ± 1.08 days). Garut ewes exhibited the highest litter size (1.77 ± 0.18; p < 0.01), surpassing both F1 Dorper × Garut crossbreds (1.33 ± 0.04) and Dorper ewes (1.30 ± 0.2). Additionally, Garut ewes achieved the highest reproduction index (2.72 ± 0.88 lambs/year), significantly exceeding F1 Dorper × Garut crossbred (1.93 ± 0.13) and Dorper ewes (1.84 ± 0.22). F1 Dorper × Garut crossbreeding enhances sheep reproductive efficiency by approaching Garut ewes for the lambing interval but not for the litter size variable.

**Table 2:** Reproductive performance of F1 Dorper × Garut crossbred, Garut, and Dorper ewes (mean ± SEM).

Variable	Ewe breed		

	F1 Garut × Dorper crossbred	Garut	Dorper
Lambing interval (day)	206.65 ± 2.75^a^	209.10 ± 1.08^a^	265.66 ± 1.14^b^
Litter size (head)	1.33 ± 0.04^a^	1.77 ± 0.18^b^	1.30 ± 0.42^a^
Mortality (%)	5.88 ± 2.80	5.53 ± 1.35	8.33 ± 5.62
Birth weight (kg)	2.70 ± 0.07^a^	2.62 ± 0.01^a^	3.35 ± 0.07^b^
Sex			
Male	2.78 ± 0.88^a^	2.64 ± 0.16^a^	3.42 ± 0.10^b^
Female	2.67 ± 0.09^a^	2.56 ± 0.02^a^	3.26 ± 0.09^b^
Birth type			
Single	2.99 ± 0.90^a^	3.07 ± 0.03^a^	3.54 ± 0.91^b^
Multiple	2.28 ± 0.07^a^	2.42 ± 0.02^a^	3.12 ± 0.09^b^
Weaning weight (kg)	19.48 ± 0.35^b^	15.36 ± 0.10^a^	23.93 ± 0.57^c^
Sex			
Male	20.27 ± 0.53^b^	16.09 ± 0.14^a^	23.75 ± 0.81^c^
Female	18.80 ± 0.45^b^	14.54 ± 0.13^a^	24.03 ± 0.76^c^
Birth type			
Single	19.24 ± 0.45^b^	15.51 ± 0.13^a^	24.59 ± 0.76^c^
Multiple	19.83 ± 0.54^b^	15.14 ± 0.14^a^	23.44 ± 0.56^c^
Average daily gain (g/day)	165.34 ± 2.95^b^	130.47 ± 0.83^a^	203.88 ± 4.65^c^
Sex			
Male	171.67 ± 4.44^b^	136.64 ± 1.18^a^	204.09 ± 7.01^c^
Female	159.90 ± 3.90^b^	123.60 ± 1.14^a^	203.75 ± 6.14^c^
Birth type			
Single	167.30 ± 3.95^b^	134.86 ± 1.13^a^	213.5 ± 6.57^c^
Multiple	162.54 ± 4.41^b^	124.24 ± 1.19^a^	198.96 ± 6.13^c^
RI (lambs/year)	1.93 ± 0.13^a^	2.72 ± 0.88^b^	1.84 ± 0.22^a^
Productivity index (kg/year)	40.83 ± 3.32	44.16 ± 2.70	50.09 ± 7.02

^a,b,c^Different superscripts in the same row indicate significant differences (p < 0.05). SEM = Standard error of mean, RI = Reproduction index

### Growth performance

Growth performance analysis revealed the superior growth potential of Dorper lambs, which exhibited significantly higher (p < 0.05) birth weight (3.35 ± 0.07 kg), weaning weight (23.93 ± 0.57 kg), and pre-weaning ADG (203.88 ± 4.65 g/day). While Garut lambs demonstrated the lowest growth metrics, F1 Dorper × Garut crossbreds showed significant (p < 0.05) improvement over Garut lambs in birth weight (2.70 ± 0.07 kg vs. 2.62 ± 0.01 kg), weaning weight (19.48 ± 0.35 kg vs. 15.36 ± 0.10 kg), and ADG (165.34 ± 2.95 g/day vs. 130.47 ± 0.83 g/day) (Table 2). These findings highlight that F1 Dorper × Garut crossbreds are promising candidates for meat production and optimizing lamb production, with a focus on rapid growth.

### Comparison with other breeds

As summarized in Table 2, Dorper lambs consistently exhibited superior pre-weaning growth performance (p < 0.05), with significantly higher birth weight, pre-weaning ADG, and weaning weight compared with both F1 Dorper × Garut crossbreds and pure Garut lambs. Although birth weights did not differ significantly between F1 Dorper × Garut crossbreds and Garut lambs, the F1 Dorper × Garut crossbreds exhibited significantly improved (p < 0.05) postnatal growth, as reflected in higher ADG (165.34 ± 2.95 g/day vs. 130.47 ± 0.83 g/day) and weaning weight (19.48 ± 0.35 kg vs. 15.36 ± 0.10 kg). This advantage was observed across sexes and birth types (single vs. multiple), highlighting the potential of Dorper × Garut crossbreeding to enhance growth traits while retaining the local Garut breed’s adaptive characteristics.

## DISCUSSION

### Variation in reproductive traits

The reproductive performance of the purebred Dorper, Garut, and their crossbreeds demonstrated significant differences in key reproductive traits (Table 2). Lambing interval, litter size, and reproductive index varied among breeds (p < 0.05). The lambing interval of F1 Dorper × Garut crossbred ewes (206.65 ± 2.75 days) closely resembled that of purebred Garut ewes (209.10 ± 1.08 days) but was significantly shorter than that of purebred Dorper ewes (265.66 ± 1.14 days). These results corroborate previous findings that Dorper sheep generally exhibit longer lambing intervals than local breeds [19].

### Litter size and reproductive index

The litter size did not differ significantly between F1 Dorper × Garut crossbreds (1.33 ± 0.04) and purebred Dorper ewes (1.30 ± 0.42), although both litter sizes were significantly lower than those of purebred Garut ewes (1.77 ± 0.18). These findings are consistent with those of the existing literature, suggesting a trade-off between prolificacy and growth performance in crossbred sheep [20, 21]. Additionally, the reproductive index was highest in Garut ewes (2.72 ± 0.88), followed by F1 Dorper × Garut crossbreds (1.93 ± 0.13) and Dorper ewes (1.84 ± 0.22). The superior reproductive index of Garut ewes underscores their high prolificacy, which is an essential characteristic of local breeding programs.

The genetic predisposition of Garut sheep to high litter sizes is a well-documented trait among prolific local breeds [22]. This prolific trait is associated with genetic factors and adaptation to tropical environments, where local sheep, such as Garut, have evolved with high ovulation rates and good maternal ability. Genetically, Garut sheep exhibit polymorphisms in the growth differentiation factor 9 (*GDF9*) gene, with the single-nucleotide polymorphism g.333G>A significantly associated with litter size [23]. This confirms that the high prolificacy of Garut sheep can be traced at the molecular level, highlighting the potential use of genetic markers in future breeding programs. Conversely, Dorper sheep, primarily selected for growth performance, typically produce smaller litters, consistent with previous crossbreeding studies by Deng *et al*. [24] and Tesema *et al*. [25]. Environmental factors, management practices, and overall flock health likely contributed to the observed variability in litter size among Garut ewes [26].

### Trade-offs between reproduction and growth

These results also indicate a trade-off between reproductive performance and growth among the three sheep breeds. Garut sheep, which exhibited a higher litter size and reproductive index, showed lower birth and weaning weights. Conversely, Dorper and Dorper × Garut crossbred (F1) sheep exhibited higher growth performance but produced fewer offspring. This inverse relationship aligns with the biological principle that increasing litter size may reduce nutrient availability per individual, thereby decreasing birth and weaning weights. Because of the absence of nutrient competition during gestation and lactation, single-born lambs have higher birth weights than twins [27]. Therefore, to achieve optimal outcomes in local farming systems, breeding strategies should consider balancing growth and reproductive traits.

### Birth weight and growth variation

The birth weight, a crucial determinant of early growth and survival, also exhibited significant variation among breeds. Dorper lambs had the highest birth weight (3.35 ± 0.07 kg), which was significantly greater than that of Garut lambs (2.62 ± 0.01 kg) and F1 Dorper × Garut crossbred lambs (2.70 ± 0.07 kg) (Table 2). These findings are consistent with a previous study by Ayichew [28], which highlights the genetic superiority of Dorper sheep in terms of growth traits. The lower birth weight in Garut lambs is likely a consequence of their larger litter sizes, as intrauterine competition for nutrients generally results in reduced individual birth weights [29]. Similar studies have reported that increased litter size correlates with lower birth weight due to maternal resource limitations [30, 31].

The birth weight of F1 Garut-Dorper crossbred lambs (2.70 ± 0.98 kg) closely aligns with the previous studies (Table 3) [6, 8, 11, 20, 22, 29, 32–38]. However, the birth weight of Dorper lambs in this study was lower than reported values from African populations (3.80 ± 0.8 kg) [37, 39], although comparable to those observed in similar climatic conditions (3.04 ± 0.04 kg) [9]. This variation highlights the influence of environmental factors on lamb growth, including temperature, nutrition, and management.

**Table 3:** Birth weight, weaning weight, and ADG of lambs across different local sheep breeds, Dorpers, and their crossbreeds (mean ± SD).

Breed	Sex	Birth weight (kg)	Weaning weight (kg)	ADG (g/day)	Test days	References
Garut	Male and female	2.62 ± 0.69	15.36 ± 5.18	130.47 ± 44.35	90	This study
Dorper purebred	Male and female	3.35 ± 0.84	23.93 ± 6.91	203.88 ± 56.56	90	This study
F1 Dorper × Garut	Male and female	2.70 ± 0.98	19.48 ± 5.52	165.34 ± 46.91	90	This study
Dorper × Garut	Male and female	2.70 ± 0.71	16.01 ± 0.71	145.79 ± 33.22	90	[6]
Garut × Garut	Male and female	2.21 ± 0.53	14.15 ± 2.94	109.35 ± 27.96	90	[6]
F1 Namaqua Afrikaner × Dorper	Male and female	4.40 ± 0.11	33.10 ± 0.70	-	-	[8]
Dorper	Male and female	4.19 ± 0.09	32.70 ± 0.60	-	-	[8]
Namaqua Afrikaner	Male and female	3.96 ± 0.10	26.80 ± 0.60	-	-	[8]
Dorper crossbreed	Male and female	3.32	15.30	-	-	[11]
Garut	Male and female	2.40 ± 0.68	9.67 ± 0,28	-	90	[32]
Local (Eastern Amhara)	Male and female	2.36 ± 0.05	8.53 ± 0.14	67.78 ± 1.60	90	[20]
Dorper × local crossbred	Male and female	3.24 ± 0.04	14.95 ± 0.21	129.97 ± 2.23	90	[20]
Dorper × Tumele	Male and female	3.29	13.70	115.3	90	[22]
Dorper × Tumele	Male	3.28	13.90	116.8	90	[22]
Dorper × Tumele	Female	3.30	13.60	113.9	90	[22]
Dorper purebred	Male and female	3.39 ± 0.08	16.18 ± 0.35	142.93 ± 3.89	85-95	[29]
Dorper × Afar	Male and female	2.57 ± 0.06	9.45 ± 0.87	73.19 ± 10.89	85-95	[29]
Dorper × Menz	Male and female	2.77 ± 0.04	12.34 ± 0.25	106.24 ± 2.61	85-95	[29]
Pelibuey	Male and female	3.27 ± 0.12	10.72 ± 0.35	130.00	56	[33]
Katahdin	Male and female	3.95 ± 0.14	11.28 ± 0.41	130	56	[33]
F1 Katahdin × East Friesian	Male	3.90	19.03	152.86	90	[34]
F1 Katahdin × East Friesian	Female	3.60	15.74	127.01	90	[34]
Romanov	Male and female	3.89	22.16	202.00	94.36	[35]
Katahdin	Male and female	4.07	23.47	212	91.50	[35]
Dorper	Male and female	4.43	24.75	219	88.54	[35]
F1 Perendale crossbred cattle	Male and female	2.33 ± 0.12	10.14 ± 0.51	85.80 ± 5.04	90	[36]
F1 crossbred Dorper	Male and female	2.37 ± 0.13	10.24 ± 0.56	86.43 ± 5.51	90	[36]
F1 Damara crossbred cattle	Male and female	2.12 ± 0.09	10.30 ± 0.39	89.89 ± 3.92	90	[36]
F1 BNS	Male and female	1.68 ± 0.84	7.83 ± 0.37	67.67 ± 3.66	90	[36]
F1 Dorper × Harargle Highland	Male and female	3.00 ± 0.10	15.10 ± 0.30	100.40 ± 2.60	120	[37]
F1 Dorper × Blackhead Ogaden	Male and female	2.90 ± 0.10	14.90 ± 0.10	99.20 ± 2.20	120	[37]
Dorper purebred	Male and female	3.79 ± 0.02	17.47 ± 0.14	-	90	[38]
Red Maasai purebred	Male and female	3.15 ± 0.02	14.71 ± 0.13	-	90	[38]
F1 Dorper × Red Maasai	Male and female	3.43 ± 0.02	16.61 ± 0.11	-	90	[38]

ADG = Average daily gain, SD = Standard deviation, BNS = BLRI Native Sheep

### Mortality and lamb viability

Importantly, no significant differences in mortality rates were detected across breeds, which are consistent with the findings of Lakew *et al*. [20], who reported that sire breed had a minimal impact on lamb survival. This suggests that external factors such as housing, nutrition, and healthcare management play a more substantial role in lamb viability than genetic background alone.

### Evidence from previous crossbreeding studies

Previous studies by Athifa *et al*. [6], Gebreyowhens *et al*. [38], and Teklebrhan *et al*. [40] have demonstrated that crossbreeding with Dorper rams enhances pre-weaning growth performance in lambs. Dorper crossbreeding can improve production performance by up to 53% in local sheep [38]. For instance, pre-weaning performance in indigenous Blackhead Ogaden and Hararghe Highland sheep improved with 50%–75% Dorper inheritance [40]. Similarly, crossbred lambs from indigenous Namaqua Afrikaner and Dorper showed a 7.9% increase in birth weight and an 11.2% increase in weaning weight compared with purebred counterparts [8].

### Role of heterosis in growth performance

In this context, the improved growth performance of F1 Dorper × Garut lambs compared with purebred Garut lambs reflects the expression of heterosis, particularly in early growth traits. Crossbreeding between genetically distinct breeds, such as Dorper and Garut, allows for hybrid vigor through complementary genetic contributions. The increased weaning weight of F1 lambs relative to Garut lambs supports the potential genetic advantage of crossbreeding. This heterotic effect can be strategically utilized to enhance production efficiency in local farming systems.

### Influence of sex, birth type, and environment

Pre-weaning growth is also influenced by sex and birth type, with single-born and male lambs typically outperforming multiples and females [40]. Although structured breeding programs can enhance local sheep genetics, crossbreeding with Dorper is not recommended for harsh environments due to differences in adaptability [41]. Continuous backcrossing with first-generation crossbred males may improve local adaptability [38]. In addition, lamb growth and performance are shaped by management practices, nutrition, environmental conditions, and breed-specific traits [42].

### Practical implications for sheep breeding

Overall, this study provides valuable insights into the reproductive and growth performance of F1 Dorper × Garut crossbred sheep. Although crossbreeding with Dorper rams offers potential for improved growth performance, reproductive traits and environmental adaptability should be integrated into breeding strategies to ensure sustainable productivity in local sheep farming systems.

A comparison of the reproductive performance of ewes and the growth of lambs before weaning in purebred Dorper, Garut, and their crossbred sheep shows that crossbreeding can be a promising alternative for improving the productivity of local sheep. Therefore, the results of this study have important implications for the development of sheep breeding strategies in Indonesia, such as maintaining pure Garut sheep flocks for breeding purposes and utilizing the first generation of Dorper × Garut crossbreeds for commercial fattening purposes. Given that this study is limited to the maternal reproduction and growth of lambs before weaning, further research is needed to comprehensively assess the sustainability and benefits of this crossbreeding program.

## CONCLUSION

This study provides clear evidence that crossbreeding Dorper and Garut sheep can strategically combine the reproductive advantages of the local Garut breed with the growth potential of Dorper. The results demonstrated that F1 Dorper × Garut crossbred ewes had significantly shorter lambing intervals (206.65 ± 2.75 days) than purebred Dorper ewes (265.66 ± 1.14 days), resembling those of Garut ewes (209.10 ± 1.08 days). However, Garut ewes maintained superiority in litter size (1.77 ± 0.18) and reproductive index (2.72 ± 0.88 lambs/year), compared with both F1 crossbreds (1.33 ± 0.04; 1.93 ± 0.13) and Dorper ewes (1.30 ± 0.42; 1.84 ± 0.22). In terms of growth performance, Dorper lambs excelled in birth weight (3.35 ± 0.07 kg), weaning weight (23.93 ± 0.57 kg), and pre-weaning ADG (203.88 ± 4.65 g/day). Importantly, F1 Dorper × Garut lambs showed significant improvements over Garut lambs in weaning weight (19.48 ± 0.35 vs. 15.36 ± 0.10 kg) and ADG (165.34 ± 2.95 vs. 130.47 ± 0.83 g/day), highlighting their promise for meat production in tropical conditions.

The findings support the integration of F1 Dorper × Garut crossbreds into Indonesian sheep production systems, where they can serve as a balance between reproductive efficiency and improved growth rates. A dual strategy is recommended: maintaining pure Garut flocks as breeding stock for their high prolificacy and adaptability, while utilizing F1 Dorper × Garut crossbreds in commercial fattening programs to meet rising meat demand. This approach provides a practical model for enhancing productivity and sustaining local genetic resources.

The research is based on a large-scale, field-based dataset encompassing 1,744 ewes and 3,248 lambs, making it one of the most comprehensive evaluations of Dorper × Garut crossbreeding under commercial conditions in Indonesia. The inclusion of multiple reproductive and growth parameters strengthens the reliability and applicability of the results to real-world production systems.

The study focused primarily on maternal reproductive traits and pre-weaning growth performance. It did not extend to post-weaning growth, carcass characteristics, long-term adaptability, or the economic profitability of crossbreeding systems. Moreover, environmental and nutritional influences, though controlled, may still have contributed to variation across groups.

Future research should investigate multigenerational crossbreeding schemes, carcass yield and meat quality traits, and the economic feasibility of scaling up Dorper × Garut programs under commercial production systems. Molecular genetic studies, particularly the use of marker-assisted selection (e.g., GDF9 polymorphisms), could further optimize breeding strategies. Additionally, evaluation under diverse agro-ecological zones is warranted to assess adaptability and resilience.

In conclusion, Dorper × Garut crossbreeding represents a promising pathway to enhance sheep productivity in Indonesia, enabling a synergy between local adaptability and improved growth performance. With careful integration of reproductive and growth traits, coupled with evidence-based breeding policies, this crossbreeding strategy can contribute to sustainable livestock development, improved rural livelihoods, and enhanced national meat security.

## AUTHORS’ CONTRIBUTIONS

ZNJ: Data curation, investigation, methodology, formal analysis, visualization, writing – original draft, and writing – review and editing. PP: Conceptualization, supervision, funding acquisition, validation, and writing - review and editing. AHKA: Formal analysis, methodology, software, writing – original draft, and writing – review and editing. BAA: Conceptualization, project administration, data curation, validation, writing - original draft, and writing - review and editing. SA: Formal analysis, validation, writing – original draft, and writing – review and editing. ATW: Validation, writing – original draft, and writing – review and editing. MFH: Data curation and investigation. BTNH: Data curation and visualization. AS: Validation and writing - review and editing. AI: Conceptualization, methodology, data curation, formal analysis, resources, writing - original draft, and writing - review and editing. All authors have read and approved the final manuscript.
